# Predictive value of preoperative inflammatory response biomarkers for metabolic syndrome and post-PCNL systemic inflammatory response syndrome in patients with nephrolithiasis

**DOI:** 10.18632/oncotarget.20344

**Published:** 2017-08-18

**Authors:** Kun Tang, Haoran Liu, Kehua Jiang, Tao Ye, Libin Yan, Peijun Liu, Ding Xia, Zhiqiang Chen, Hua Xu, Zhangqun Ye

**Affiliations:** ^1^ Department of Urology, Tongji Hospital, Tongji Medical College, Huazhong University of Science and Technology, Wuhan 430030, China; ^2^ Institute of Urology of Hubei Province, Wuhan 430030, China

**Keywords:** inflammatory response biomarkers, nephrolithiasis, percutaneous nephrolithotomy, metabolic syndrome, systemic inflammatory response syndrome

## Abstract

Neutrophil to lymphocyte ratio (NLR), derived neutrophil to lymphocyte ratio (dNLR), platelet to lymphocyte ratio (PLR) and lymphocyte to monocyte ratio (LMR) were promising biomarkers used to predict diagnosis and prognosis in various inflammatory responses diseases and cancers. However, there have been no reports regarding these biomarkers in kidney stone patients. This study aimed to evaluate the predictive value of inflammatory biomarkers for metabolic syndrome (MetS) and post-PCNL SIRS in nephrolithiasis patients. We retrospectively enrolled 513 patients with nephrolithiasis and 204 healthy controls. NLR, dNLR, LMR and PLR in nephrolithiasis patients were significantly higher than control group. Patients with renal stone have higher NLR, dNLR, LMR and PLR than those without. ROC curve analysis indicated NLR, dNLR, LMR and PLR for predicting patients with nephrolithiasis and MetS, displayed AUC of 0.730, 0.717, 0.627 and 0.606. Additionally, ROC curves, using post-PCNL SIRS as the end-point for NLR, dNLR, LMR and PLR with AUC of 0.831, 0.813, 0.723 and 0.685. Multivariate analysis revealed that NLR, dNLR represented independent factors for predicting post-PCNL SIRS. While LMR independently associated with MetS. These resluts demonstrate preoperative NLR, dNLR and LMR appears to be effective predictors of post-PCNL SIRS and LMR of MetS in nephrolithiasis patients.

## INTRODUCTION

Nephrolithiasis is an increasingly common condition in the Asia with a prevalence of 10.6% and 7.1% in men and women, respectively, [1] thus causing a heavy economic burden. Additionally, there is an increased risk for patients with kidney stones to stones recurrence, chronic kidney disease (CKD), diabetes, cardiovascular diseases and renal cancer. Previous studies have identified some lifestyle factors, including MetS, as a risk factor for kidney stone formation and contributors to the recurrence of kidney stones.

Several results showed that inflammation plays a significant part in the initial stage and progression of kidney stones [2]. Besides local inflammatory diseases, a portion of patients with cancers can appear systemic inflammatory responses, which are characterized by changes of peripheral blood cell counts. Therefore, several circulating blood cell-based biomarkers including: neutrophil to lymphocyte ratio (NLR), derived neutrophil to lymphocyte ratio (dNLR), [3] platelet to lymphocyte ratio (PLR) and lymphocyte to monocyte ratio (LMR) - were used to predict diagnosis and prognosis in various diseases. These markers can be readily calculated from routine complete blood counts from peripheral blood specimens in the clinical practice. Furthermore, they are routinely measured and inexpensive to test, and hence potentially provide readily available objective information to help doctors to estimate patient progression.

The relationship between inflammatory biomarkers and various diseases has been demonstrated by numerous studies, highlighting the potential of preoperative inflammatory biomarkers [4–8]. However, to date, there have been no reports regarding NLR in kidney stone patients.

**Figure 1:**
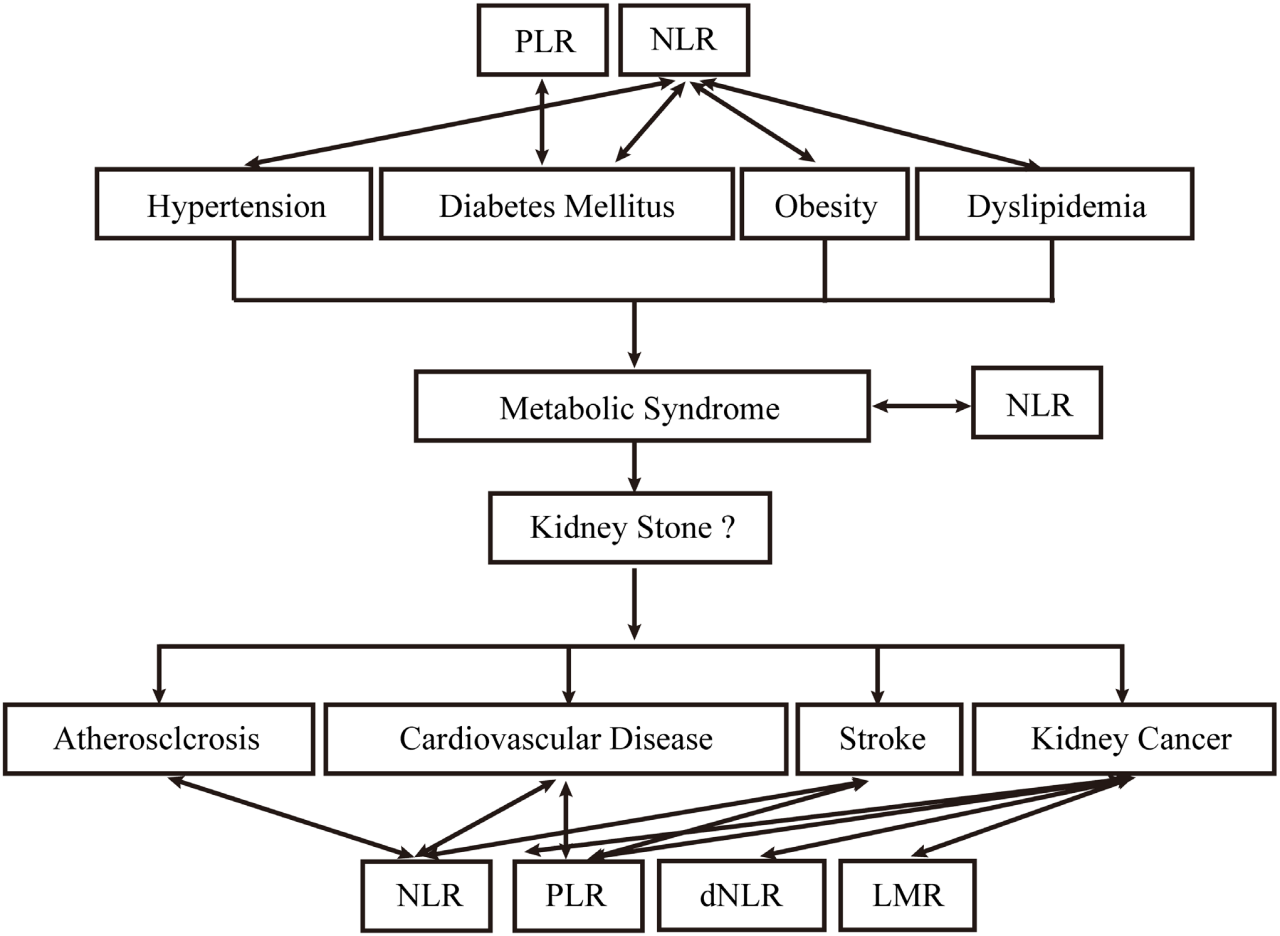
Etiological and complicated diseases of kidney stone and their association with NLR, dNLR, LMR and PLR

Previous epidemiological studies have shown an increased prevalence of kidney stones in patients with lifestyle-related systemic diseases such as hypertension [9]. diabetes mellitus, [10] obesity [11] and dyslipidemia. Taken together, these risk factors are termed metabolic syndrome (MetS). Furthermore, associations between nephrolithiasis and atherosclerosis, [12] cardiovascular disease, [13] renal cell carcinoma [14] have been recognized, and patients are at risk of these diseases after stone formation (Figure 1).

Interestingly, preoperative inflammatory response biomarkers, such as NLR, are considered to be strong predictors of these diseases, including hypertension, [4] obesity, [5] diabetes mellitus, [6] dyslipidemia, [15] MetS, [5, 8, 16] atherosclerosis, [17] cardiovascular disease, [18] stroke, [19] and renal carcinoma [7]. Aggregate evidence suggests kidney stone formation becomes increasingly prevalent in people with MetS [20–22]. This association seems to be reciprocal in nature as patients with kidney stones seem to harbor MetS and ones with MetS are at increased risk for kidney stones (Figure 1) [12]. Owing to the complex causal relationship between MetS and nephrolithiasis, we investigated the predictive role of inflammation biomarkers for MetS-comorbidities in kidney stones

Percutaneous nephrolithotomy (PCNL) is one of the most common treatments for large renal and upper ureteric calculi [23]. For patients undergoing PCNL, there is a high possibility of developing systemic inflammatory response syndrome (SIRS), bacteremia and urosepsis [24]. Up to 35% of patients with complicated stones may develop SIRS, with a small percent progressing to sepsis [13]. Notably, in large cohort studies, sepsis was reported to be the most common cause of perioperative mortality following PCNL [25]. Previous researches reported that stone size, stone culture, and pelvic urine culture are crucial risk factors for SIRS following PCNL [23]. The present clinical parameters for predicting the post-PCNL SIRS infection are white cell counts, C-reaction protein (CRP) and procalcitonin (PCT), however, CRP and PCT are not routinely tested in our clinical practice, especially in the context of preoperative testing. And the pre-treatment white cell counts had limited role in predicting the post-PCNL SIRS.

We hypothesized the elevated NLR in patients with kidney stone, especially for patients with MetS comorbidities, and could play as a potential predictor of post-PCNL SIRS. However, the association between kidney stone and preoperative inflammatory response biomarkers, such as NLR, dNLR, LMR and PLR still remained unknown. The current research thus aimed to evaluate the clinical significance of the NLR, dNLR, LMR and PLR for detecting MetS, SIRS and to determine whether these biomarkers could serve as a predictor for MetS comorbidities and post-PCNL SIRS in patients with kidney stones.

## RESULTS

### Basic characteristics of the study sample

The clinicopathological characteristics of patients and healthy controls are presented in Table 1. Patients with kidney stones had a median age of 52 years (range: 19–84), with a gender ratio of 1.75:1 men to women. The median age in the healthy control group was 51 years (range: 19–79) with a gender ratio of 1.7:1 (men:women) Three were no statistically significant differences between the age and gender of the two groups (P > 0.05).

**Table 1:** Basic characteristics of the study sample

Variables	Kideny stone formers N=513	Healthy controls N=204	P value
NLR	2.31 ± 1.70	1.61 ± 0.56	< 0.001
dNLR	1.59 ± 0.98	1.22 ± 0.40	< 0.001
LMR	4.17 ± 1.60	5.09 ± 1.63	< 0.001
PLR	122.90 ± 57.21	126.37 ± 52.37	0.454
Age, (years)	52.27 ± 10.95	50.81 ± 11.91	0.118
Gender, (male%)	294 (57.31%)	120 (58.5)	0.731
BMI, (kg/m^2^)	23.2 ± 3.08	22.418 ± 3	< 0.001
Hypertension, n (%)	43 (8.4%)	0 (0%)	< 0.001
KillipI, n (%)	487 (94.3%)	204 (100%)	< 0.001
KillipII, n (%)	22 (4.3%)	0 (0%)	
KillipIII, n (%)	4 (0.7%)	0 (0%)	
KillipIV, n (%)	0 (0%)	0 (0%)	
Diabetes, n (%)	87 (20%)	0 (0%)	< 0.001
ASA1, n (%)	218 (42.5%)	151 (74%)	< 0.001
ASA2, n (%)	265 (51.7%)	53 (26%)	
ASA3, n (%)	30 (5.8%)	0 (0%)	
White blood cell, (10^9^/L)	6.12 ± 1.65	5.98 ± 1.32	0.270
Neutrophil, (10^9^/L)	3.66 ± 1.39	3.23 ± 0.99	< 0.001
Lymphocyte, (10^9^/L)	1.8 ± 0.55	2.12 ± 0.57	< 0.001
Monocyte, (10^9^/L)	0.48 ± 0.19	0.43 ± 0.12	0.009
Platelet, (10^9^/L)	201.92 ± 64.04	249.95 ± 77.48	< 0.001
Hemoglobin, (g/L)	126.11 ± 22.4	138.38 ± 26.78	< 0.001
Alkaline phosphatase, (IU/L)	69.61 ± 23.45	59.34 ± 18.49	< 0.001
Albumin, (g/L)	40.99 ± 26.55	42.48 ± 3.76	0.425
Total Cholesterol, (mmol/L)	4.07 ± 0.82	4.09 ± 0.67	0.714
FBG, (mmol/L)	5.33 ± 1.24	4.89 ± 0.64	< 0.001
Sodium, (mmol/L)	140.76 ± 4.14	141.92 ± 2.15	< 0.001
Chloride, (mmol/L)	105.06 ± 3.60	104.41 ± 2.20	0.015
Calcium, (mmol/L)	2.39 ± 1.70	2.32 ± 0.13	0.553
Potassium, (mmol/L)	4.65 ± 8.47	4.29 ± 0.39	0.540
Bicarbonate, (mmol/L)	24.24 ± 4.72	24.91 ± 2.53	0.056
Uric acid, (umol/L)	365.53 ± 121.62	312.39 ± 76.23	< 0.001
Serum creatinine, (umol/L)	139.86 ± 152.49	77.47 ± 7.13	< 0.001
BUN, (mol/L)	7.43 ± 5.79	5.45 ± 4.16	< 0.001
GFR, (ml/min)	67.87 ± 30.39	102.03 ± 20.77	< 0.001

NLR, neutrophil to lymphocyte ratio; dNLR, neutrophil to (white cell count- neutrophil count) ratio; LMR, lymphocyte to monocyte ratio; PLR, platelet count to lymphocyte ratio; BMI, body mass index; ASA, American Society of Anesthesiologists; FBG, fasting blood-glucose; BUN, blood urea nitrogen; GFR, glomerular filtration rate.

Table 1 also shows the laboratory and clinical characteristics of the groups. Levels of diabetes, ASA score, neutrophil, lymphocyte, monocyte, platelet, hemoglobin, alkaline phosphatase, FBG, sodium, chloride, uric acid, serum creatinine, BUN and GFR were significantly different in kidney stone group compared with the healthy control group (all P < 0.05). In terms of white blood cell counts, albumin, total cholesterol, calcium, potassium and bicarbonate, no significant differences were found between the two groups.

### NLR, dNLR, LMR and PLR levels were increased in kidney stone patients

**Figure 2:**
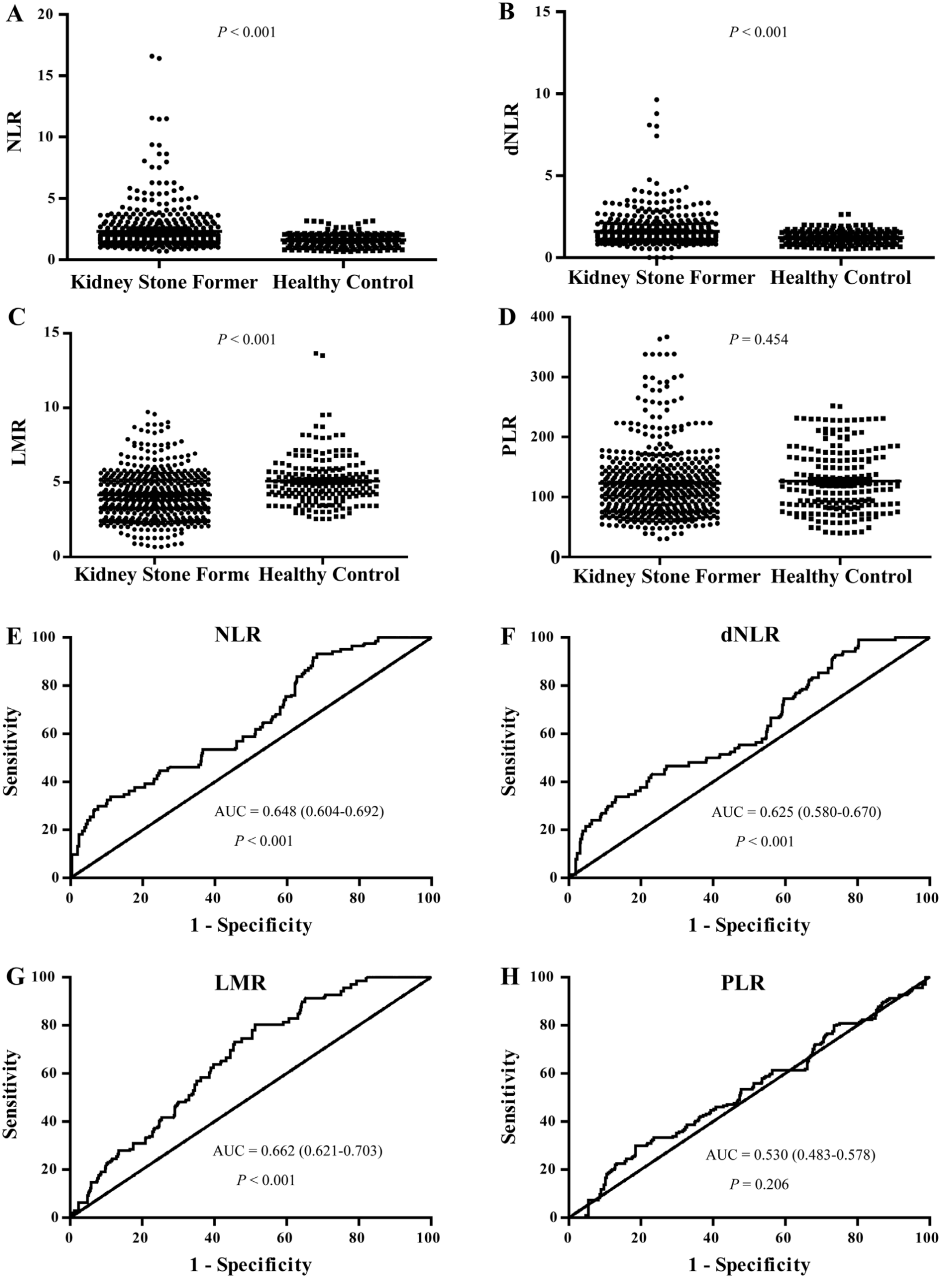
NLR, dNLR, LMR and PLR levels in kidney stone patients and their ROC curves for the identification of kidney stone patients **(A, B, C, D)** showed the NLR, dNLR, LMR and PLR levels in kidney stone patients compared with the healthy control group, respectively. **(E, F, G, H)** showed the ROC curves of NLR, dNLR, LMR and PLR for the identification of kidney stone patients, respectively.

There was a statistically significant difference in NLR, dNLR and LMR between the patients with kidney stones and healthy controls (all P < 0.001), while the PLR was found to be comparable between the two groups. We found that patients with kidney stones had higher NLR and dNLR than the healthy controls (P < 0.001) (Table 1, Figure 2). The kidney stone patients also had significantly lower LMR level (P < 0.001) (Table 1, Figure 2).

### ROC curves of NLR, dNLR, LMR and PLR for the identification of kidney stone patients from healthy control

ROC curve analysis suggested that NLR, dNLR and LMR had limited utility for the identification of patients with kidney stones based on area under the curve (AUC) values of 0.648 [95% confidence interval (CI): 0.604–0.692], 0.625 (0.58–0.67), 0.662 (0.621–0.703) respectively (Figure 2, P < 0.001). PLR could not identify patients with kidney stone patients from healthy controls (AUC 0.53 (0.483–0.578), P = 0.206 (Figure 2).

### NLR, dNLR, LMR and PLR levels were increased in patients with MetS and post-PCNL SIRS

**Figure 3:**
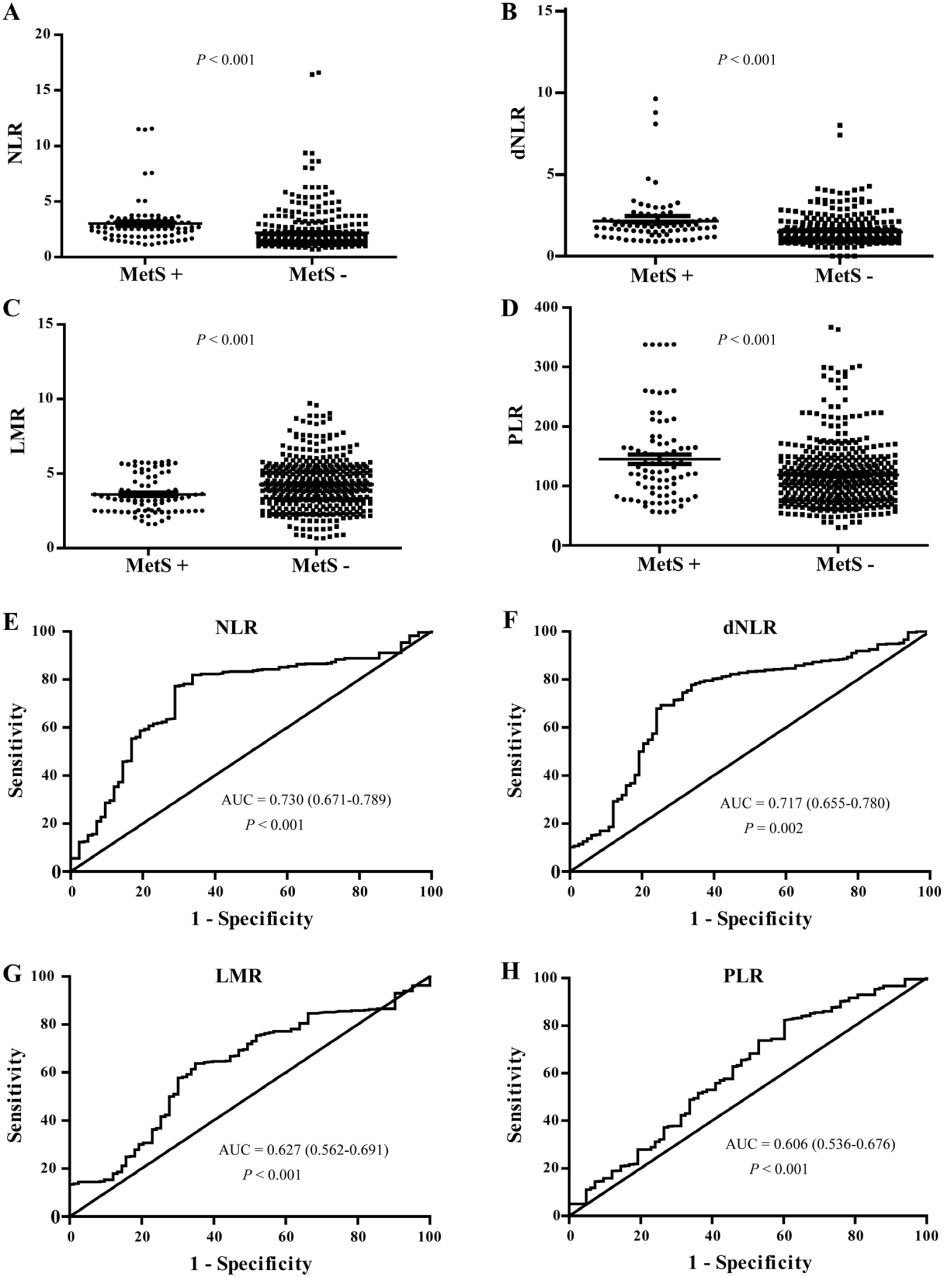
NLR, dNLR, LMR and PLR levels in kidney stone patients with and without MetS, and their ROC curves of for the predicating of MetS **(A, B, C, D)** showed the NLR, dNLR, LMR and PLR levels in kidney stone patients with or without MetS, respectively. **(E, F, G, H)** showed the ROC curves of NLR, dNLR, LMR and PLR for the predicating of MetS in kidney stone patients, respectively.

**Figure 4:**
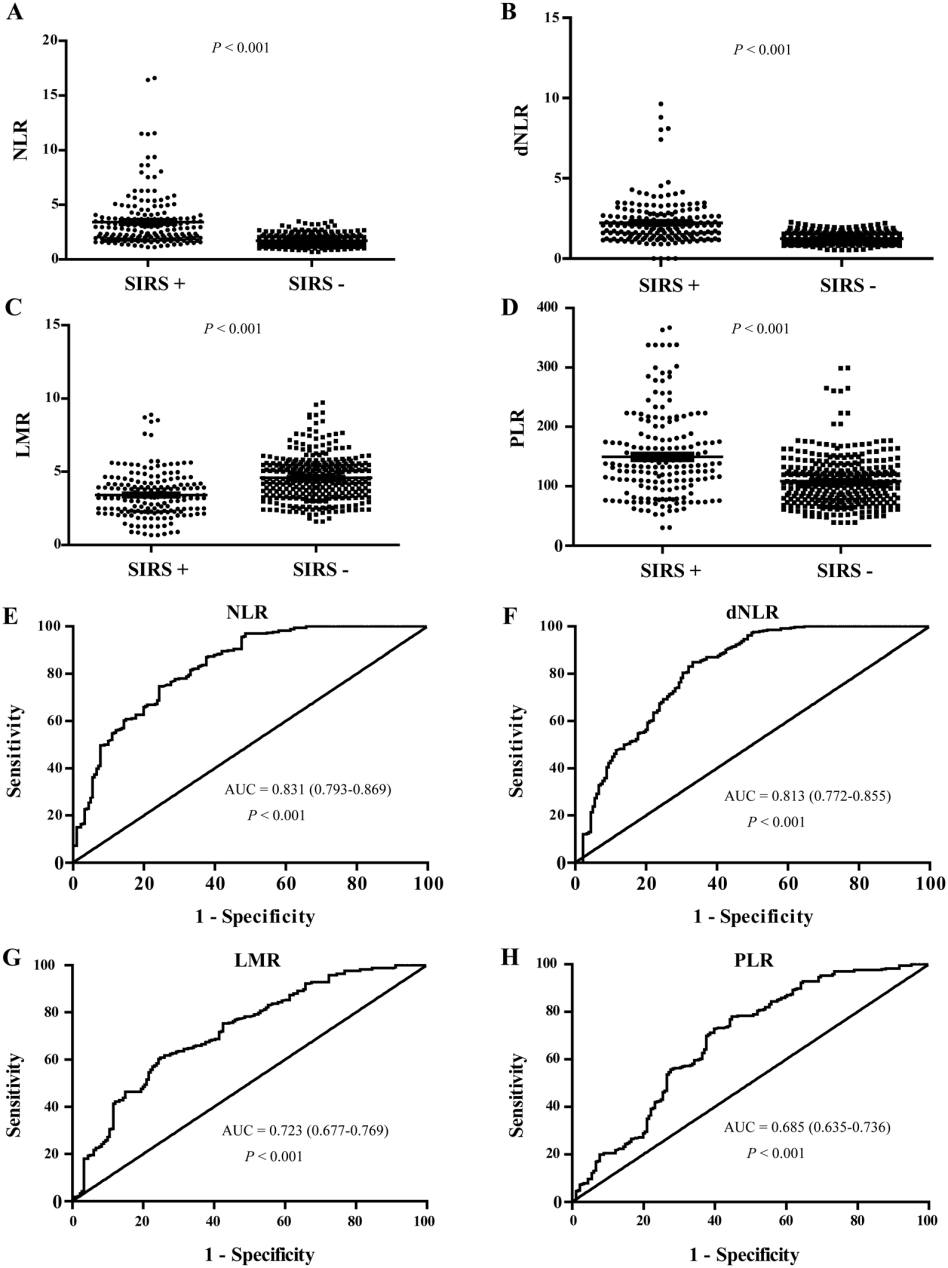
NLR, dNLR, LMR and PLR levels in kidney stone patients with and without post-PCNL SIRS, and their ROC curves for the predicating of post-PCNL SIRS **(A, B, C, D)** showed the NLR, dNLR, LMR and PLR levels in kidney stone patients with or without post-PCNL SIRS, respectively. **(E, F, G, H)** showed the ROC curves of NLR, dNLR, LMR and PLR for the predicating of post-PCNL SIRS in kidney stone patients, respectively.

There was a statistically significant difference in NLR, dNLR, LMR and PLR in patients with kidney stones with MetS and post-PCNL SIRS compared to those without (all P < 0.001). We found that the patients with kidney stones had higher NLR, dNLR and PLR than healthy controls ( P < 0.001) (Table 2, Figures 3&4), while LMR was significantly lower (P < 0.001) (Table 2, Figures 3&4).

**Table 2:** Clinical characteristics in kidney stone patients with or without SIRS and MetS

Variables	SIRS(+) N=181	SIRS(-) N=332	P value	MetS(+) N=83	MetS(-) N=430	P value
NLR	3.42 ± 2.42	1.70 ± 0.50	<0.001	3.03 ± 2.00	2.17 ± 1.61	<0.001
dNLR	2.23 ± 1.38	1.25 ± 0.33	<0.001	2.16 ± 1.50	1.49 ± 0.80	<0.001
LMR	3.42 ± 1.52	4.58 ± 1.49	<0.001	3.60 ± 1.25	4.28 ± 1.64	<0.001
PLR	149.55 ± 14.95	108.37 ± 22.54	<0.001	145.10 ± 79.55	118.61 ± 20.99	<0.001
Age, (years)	52.48 ± 11.1	52.15 ± 10.86	0.739	53.1 ± 10.2	52.1 ± 11.1	0.426
Gender, (male, %)	92 (50.83%)	202 (60.84%)	0.028	31 (37.35%)	263 (61.16%)	<0.001
BMI, (kg/m^2^)	23.1 ± 3.08	23.28 ± 3.09	0.529	25.3 ± 2.68	22.8 ± 3	<0.001
Hypertension, n (%)	32 (17.86%)	59 (17.77%)	0.979	56 (67.47%)	55 (12.79%)	<0.001
KillipI, n (%)	171 (94.48%)	312 (93.98%)	0.019	73 (87.95%)	410 (95.35%)	0.006
KillipII, n (%)	6 (3.31%)	16 (4.82%)		8 (9.64%)	14 (3.26%)	
KillipIII, n (%)	4 (2.21%)	0 (0%)		2 (2.41%)	2 (0.47%)	
Diabetes, n (%)	30 (16.57%)	57 (17.17%)	0.864	53 (63.86%)	45 (10.47%)	<0.001
ASA1, n (%)	78 (43.09%)	138 (41.57%)	0.944	14 (16.87%)	202 (46.98%)	<0.001
ASA2, n (%)	93 (51.38%)	172 (51.81%)		63 (75.90%)	202 (46.98%)	
ASA3, n (%)	10 (5.52%)	20 (6.02%)		4 (4.82%)	26 (6.05%)	
White blood cell, (10^9^/L)	6.61 ± 1.79	5.86 ± 1.5	<0.001	6.56 ± 1.85	6.04 ± 1.59	<0.001
Neutrophil, (10^9^/L)	4.45 ± 1.64	3.22 ± 1.01	<0.001	4.30 ± 1.60	3.53 ± 1.31	<0.001
Lymphocyte, (10^9^/L)	1.52 ± 0.5	1.95 ± 0.52	<0.001	1.61 ± 0.53	1.84 ± 0.55	<0.001
Monocyte, (10^9^/L)	0.51 ± 0.23	0.46 ± 0.16	0.003	0.49 ± 0.20	0.47 ± 0.19	0.531
Platelet, (10^9^/L)	202.82 ± 66.01	201.43 ± 63.03	0.814	211.40 ± 41.72	200.09 ± 60.66	0.141
Hemoglobin, (g/L)	120.7 ± 24.08	129.06 ± 20.88	<0.001	128.93 ± 20.72	125.57 ± 22.69	0.212
Alkaline phosphatase, (IU/L)	68.68 ± 25.38	70.11 ± 22.35	0.511	65.61 ± 21.55	70.38 ± 23.74	0.090
Albumin, (g/L)	37.91 ± 5.45	42.67 ± 32.65	0.053	38.21 ± 4.26	41.53 ± 28.91	0.297
Total Cholesterol, (mmol/L)	3.93 ± 0.30	4.15 ± 0.84	0.004	4.23 ± 0.78	4.04 ± 0.82	0.050
FBG, (mmol/L)	5.37 ± 1.05	5.31 ± 1.34	0.595	6.12 ± 1.65	5.18 ± 1.09	<0.001
Sodium, (mmol/L)	140.79 ± 2.88	140.75 ± 4.7	0.926	141.42 ± 2.93	140.64 ± 4.33	0.116
Chloride, (mmol/L)	105.14 ± 4.04	105.03 ± 3.34	0.731	105.04 ± 4.30	105.07 ± 3.45	0.947
Calcium, (mmol/L)	2.22 ± 0.12	2.48 ± 2.10	0.094	2.69 ± 2.85	2.33 ± 1.36	0.076
Potassium, (mmol/L)	4.24 ± 0.59	4.06 ± 0.43	0.422	4.25 ± 0.70	4.73 ± 9.	0.633
Bicarbonate, (mmol/L)	23.49 ± 5.82	24.66 ± 3.95	0.007	24.15 ± 3.62	24.26 ± 4.91	0.839
Uric acid,(umol/L)	364.19 ± 138.22	366.26 ± 111.75	0.854	376.26 ± 137.77	363.46 ± 118.32	0.380
Serum creatinine, (umol/L)	161.76 ± 182.64	127.92 ± 132.00	0.016	157.18 ± 196.62	136.52 ± 142.45	0.259
BUN, (mol/L)	8.04 ± 7.05	7.1 ± 4.95	0.078	8.13 ± 6.42	7.30 ± 5.66	0.235
GFR, (ml/min)	62.2 ± 31.69	70.97 ± 29.24	0.002	63.72 ± 29.72	68.67 ± 30.49	0.174
Stone burden, (mm^2^)	283.11 ± 248.48	204.96 ± 198.76	<0.001	253.75 ± 258.07	262.87 ± 11.78	0.881
Operative time, (minutes)	120 ± 15	117 ± 49.3	0.008	138 ± 49.5	117 ± 127	0.002
Stay length, (days)	28.28 ± 2.83	14 ± 5.5	<0.001	17 ± 2.8	15 ± 7.8	0.049
Multiple nephrolith, n (%)	171 (94.48%)	263 (80.78%)	<0.001	68 (81.93%)	373 (86.74%)	0.247
Staghorn nephrolith, n (%)	34 (25.97%)	48 (14.41%)	<0.001	18 (21.69%)	78 (18.14%)	0.448
Hydronephrosis, n (%)	177 (97.79%)	290 (87.09%)	<0.001	67 (80.72%)	400 (93.02%)	<0.001

NLR, neutrophil to lymphocyte ratio; dNLR, neutrophil to (white cell count- neutrophil count) ratio; LMR, lymphocyte to monocyte ratio; PLR, platelet count to lymphocyte ratio; BMI, body mass index; ASA, American Society of Anesthesiologists; FBG, fasting blood-glucose; BUN, blood urea nitrogen; GFR, glomerular filtration rate, SIRS, systemic inflammatory response syndrome; MetS, metabolic syndrome.

### Correlations of NLR, dNLR, LMR and PLR with clinical characteristics of kidney stone patients

In Spearman correlation analysis, there were statistically significant positive correlations between NLR, dNLR, LMR, PLR and MetS and post-PCNL SIRS (All P < 0.05) (Table 3).

**Table 3:** Correlations of NLR, dNLR, LMR and PLR with clinical characteristics of kidney stone patients

Variables	NLR		dNLR		LMR		PLR	
	R^2^	P value	R^2^	P value	R^2^	P value	R^2^	P value
Age, (years)	0.020	0.293	0.001	0.451	<0.001	0.959	0.002	0.267
Gender, (male%)	0.009	0.033	0.009	0.035	0.009	0.029	0.017	0.003
Residence, (urban)	0.003	0.206	0.002	0.330	0.004	0.175	<0.001	0.937
BMI, (kg/m2)	0.003	0.200	0.008	0.044	0.002	0.289	<0.001	0.622
Hypertension	0.001	0.999	0.002	0.273	0.006	0.084	0.002	0.306
Killip Level	0.003	0.248	0.001	0.463	0.003	0.207	0.002	0.294
Diabetes	0.020	0.001	0.030	<0.001	0.002	0.330	0.011	0.015
ASA	0.002	0.287	0.002	0.313	0.001	0.382	0.008	0.050
White blood cell, (10^9^/L)	0.166	<0.001	0.147	<0.001	0.052	<0.001	0.002	0.377
Neutrophil, (10^9^/L)	0.480	<0.001	0.467	<0.001	0.156	<0.001	0.051	<0.001
Lymphocyte, (10^9^/L)	0.287	<0.001	0.265	<0.001	0.236	<0.001	0.376	<0.001
Monocyte, (10^9^/L)	0.054	<0.001	0.007	0.062	0.389	<0.001	0.003	0.247
Platelet, (10^9^/L)	<0.001	0.877	0.001	0.474	<0.001	0.999	0.328	<0.001
Hemoglobin, (g/L)	0.044	<0.001	0.037	<0.001	0.019	0.002	0.090	<0.001
Alkaline phosphatase, (IU/L)	0.002	0.347	0.001	0.9712	<0.001	0.987	0.001	0.424
Albumin, (g/L)	0.005	0.118	0.005	0.102	0.002	0.280	0.003	0.249
Total Cholesterol, (mmol/L)	0.006	0.084	0.003	0.192	0.021	0.001	0.015	0.005
FBG, (mmol/L)	0.011	0.017	0.016	0.004	0.005	0.117	0.007	0.062
Sodium, (mmol/L)	<0.001	0.968	<0.001	0.6425	0.004	0.170	0.004	0.142
Chloride, (mmol/L)	<0.001	0.608	0.003	0.213	0.008	0.040	0.001	0.538
Calcium, (mmol/L)	0.002	0.292	0.002	0.255	0.000	0.742	0.001	0.408
Potassium, (mmol/L)	0.0013	0.424	0.001	0.445	0.001	0.563	0.003	0.209
Bicarbonate, (mmol/L)	0.002	0.313	<0.001	0.768	0.013	0.010	0.001	0.382
Uric acid, (umol/L)	0.010	0.026	<0.001	0.608	0.013	0.010	0.016	0.005
Serum creatinine, (umol/L)	0.049	<0.001	0.027	<0.001	0.038	<0.001	0.004	0.147
BUN, (mol/L)	0.045	<0.001	0.015	0.005	0.037	<0.001	<0.001	0.996
GFR, (ml/min)	0.037	<0.001	0.012	0.012	0.047	<0.001	0.003	0.256
Stone burden, (mm^2^)	0.007	0.053	0.006	0.086	0.004	0.133	0.003	0.253
SIRS	0.233	<0.001	0.228	<0.001	0.121	<0.001	0.119	<0.001
MS	0.035	<0.001	0.065	<0.001	0.025	<0.001	0.029	<0.001
Operative time, (minutes)	<0.001	0.827	<0.001	0.945	<0.001	0.677	<0.001	0.530
Stay length, (days)	0.030	<0.001	0.029	<0.001	0.004	0.176	0.011	0.020
Multiple nephrolith	0.006	0.070	0.004	0.141	0.013	0.001	0.004	0.152
Staghorn nephrolith	0.024	<0.001	0.031	<0.001	0.004	0.163	0.013	0.009
Hydronephrosis	0.005	0.120	0.003	0.214	0.008	0.039	0.002	0.350

NLR, neutrophil to lymphocyte ratio; dNLR, neutrophil to (white cell count- neutrophil count) ratio; LMR, lymphocyte to monocyte ratio; PLR, platelet count to lymphocyte ratio; BMI, body mass index; ASA, American Society of Anesthesiologists; FBG, fasting blood-glucose; BUN, blood urea nitrogen; GFR, glomerular filtration rate, SIRS, systemic inflammatory response syndrome; MetS, metabolic syndrome.

### Correlations of NLR, dNLR, LMR and PLR with MetS and post-PCNL SIRS in patients with kidney stone

In Spearman correlation analysis, there were statistically significant positive correlations between MetS and NLR, dNLR, LMR, PLR, BMI, hypertension, Killip level, diabetes, ASA score, neutrophil, lymphocyte counts, total cholesterol, FBG and operative time (Table 4).

**Table 4:** Correlations of NLR, dNLR, LMR and PLR with MetS and post-PCNL SIRS in patients with kidney stone

Variables	SIRS		P value	MetS		P value
	95% CI	R		95% CI	R	
NLR	0.411 to 0.545	0.481	<0.001	0.103 to 0.270	0.188	< 0.001
dNLR	0.405 to 0.540	0.476	<0.001	0.174 to 0.337	0.257	< 0.001
LMR	-0.420 to -0.266	-0.345	<0.001	-0.223 to -0.052	-0.139	<0.001
PLR	0.267 to 0.420	0.346	<0.001	0.081 to 0.250	0.166	<0.001
Age, (years)	-0.072 to 0.101	0.015	0.738	-0.052 to 0.121	0.035	0.426
Gender, (male%)	-0.182 to -0.010	-0.097	0.028	-0.260 to -0.092	-0.177	<0.001
Residence, (urban)	-0.151 to 0.022	-0.065	0.140	-0.123 to 0.049	-0.037	0.400
BMI, (kg/m2)	-0.114 to 0.059	-0.028	0.529	0.215 to 0.373	0.296	<0.001
Hypertension	-0.088 to 0.085	-0.001	0.979	0.055 to 0.226	0.142	<0.001
Killip Level	-0.090 to 0.083	-0.004	0.933	0.336 to 0.480	0.411	<0.001
Diabetes	-0.036 to 0.138	0.051	0.248	0.055 to 0.226	0.142	0.001
ASA	-0.112 to 0.061	-0.025	0.566	0.120 to 0.286	0.204	<0.001
White blood cell, (10^9/L)^	0.034 to 0.205	0.121	0.006	-0.125 to 0.048	-0.039	0.383
Neutrophil, (10^9^/L)	0.346 to 0.489	0.420	<0.001	0.118 to 0.284	0.203	<0.001
Lymphocyte, (10^9^/L)	-0.442 to -0.292	-0.369	<0.001	-0.236 to -0.067	-0.153	0.001
Monocyte, (10^9^/L)	0.043 to 0.213	0.129	0.003	-0.059 to 0.114	0.028	0.531
Platele,t (10^9^/L)	-0.076 to 0.097	0.010	0.814	-0.022 to 0.151	0.065	0.141
Hemoglobin, (g/L)	-0.261 to -0.094	-0.179	<0.001	-0.031 to 0.141	-0.055	0.212
Alkaline phosphatase, (IU/L)	-0.115 to 0.058	-0.029	0.511	-0.161 to 0.012	-0.075	0.090
Albumin, (g/L)	-0.171 to 0.001	-0.086	0.053	-0.132 to 0.041	-0.046	0.297
Total Cholesterol, (mmol/L)	-0.220 to -0.045	-0.133	0.004	0.000 to 0.172	0.087	0.049
FBG, (mmol/L)	0.001 to 0.177	0.089	0.043	0.198 to 0.357	0.279	<0.001
Sodium, (mmol/L)	-0.132 to 0.041	-0.002	0.2979	-0.114 to 0.060	0.028	0.536
Chloride, (mmol/L)	-0.071 to 0.102	0.015	0.731	-0.089 to 0.084	-0.003	0.947
Calcium, (mmol/L)	-0.194 to -0.018	-0.107	0.016	-0.008 to 0.164	0.079	0.076
Potassium, (mmol/L)	-0.122 to 0.051	-0.036	0.422	-0.107 to 0.0657	-0.021	0.633
Bicarbonate, (mmol/L)	-0.203 to -0.032	-0.118	0.007	-0.096 to 0.078	-0.009	0.839
Uric acid, (umol/L)	-0.095 to 0.079	-0.008	0.854	-0.048 to 0.125	0.039	0.380
Serum creatinine, (umol/L)	0.020 to 0.191	0.106	0.016	-0.037 to 0.136	0.050	0.259
BUN, (mol/L)	0.005 to 0.177	0.092	0.039	-0.034 to 0.139	0.053	0.235
GFR, (ml/min)	-0.222 to -0.052	-0.138	0.002	-0.146 to 0.027	0.060	0.174
Stone burden, (mm^2^)	0.082 to 0.251	0.028	<0.001	-0.101 to 0.073	-0.0140	0.755
Operative time, (minutes)	0.017 to 0.189	0.104	0.020	0.071 to 0.241	0.157	<0.001
Stay length, (days)	0.188 to 0.349	0.271	<0.001	-0.009 to 0.163	0.078	0.080
Multiple nephrolith	0.098 to 0.266	0.183	<0.001	-0.149 to 0.025	-0.062	0.159
Staghorn nephrolith	0.060 to 0.230	0.146	0.001	-0.062 to 0.110	0.013	0.580
Hydronephrosis	0.089 to 0.257	0.175	<0.001	-0.108 to 0.065	-0.021	0.629

NLR, neutrophil to lymphocyte ratio; dNLR, neutrophil to (white cell count- neutrophil count) ratio; LMR, lymphocyte to monocyte ratio; PLR, platelet count to lymphocyte ratio; BMI, body mass index; ASA, American Society of Anesthesiologists; FBG, fasting blood-glucose; BUN, blood urea nitrogen; GFR, glomerular filtration rate, SIRS, systemic inflammatory response syndrome; MetS, metabolic syndrome.

In addition, by spearman correlation analysis there were statistically significant positive correlations between post-PCNL SIRS and NLR, dNLR, LMR, PLR, gender, white blood cell, neutrophil, lymphocyte, monocyte counts, hemoglobin, total cholesterol, FBG, calcium, bicarbonate, serum creatinine, BUN, GFR, stone burden, operative time, stay length, multiple nephrolith, staghorn nephrolith and hydronephrosis (Table 4).

### ROC curves of NLR, dNLR, LMR and PLR for the predicating of MetS and Post-PCNL SIRS in patients with kidney stone

ROC curves, utilizing MetS as the end-point for NLR, dNLR, LMR and PLR, are depicted in Figure 3. The areas under the curve (AUC) for NLR, dNLR, PLR and LMR were 0.730, 0.717, 0.627 and 0.606, respectively (All P < 0.01).

In addition, ROC curves, using post-PCNL SIRS as the end-point for NLR, dNLR, LMR and PLR, are shown in Figure 4. The areas under the curve (AUC) for NLR, dNLR, PLR and LMR were 0.831, 0.813, 0.723 and 0.685, respectively (All P < 0.001).

### The univariate and multivariate cox analyses of NLR, dNLR, LMR and PLR for the predicating of MetS and post-PCNL SIRS in patients with kidney stone

Table 5 describes the univariate and multivariate Cox proportional hazards analyses in patients with kidney stones. Univariate Cox proportional hazards analysis, factors associated with MetS included the NLR, dNLR, LMR, PLR, BMI, hypertension, Killip level, diabetes, ASA score, White blood cell, Neutrophil, Lymphocyte counts, Total cholesterol, FBG and Operative time, Stay length, Hydronephrosis (all P < 0.05). After extensive univariate analysis, significant variables included in the multivariable Cox proportional hazards models, which showed that the level of LMR (HR 0.602, 95%CI 0.461∼0.785), BMI (HR 1.435, 95%CI 1.260∼1.633), Diabetes (HR 8.858, 95%CI 3.636∼21.579), ASA score (HR 2.552, 95%CI 1.452∼4.485), Lymphocyte (HR 0.207, 95%C: 0.048∼0.891), total cholesterol (HR 1.586, 95%CI 1.093∼2.302) and operative time (HR 1.010, 95%CI 1.004∼1.016) were significant predictors of MetS comorbidities.

**Table 5:** The univariate and multivariate Cox analyses of NLR, dNLR, LMR and PLR for the predicating of MetS and post-PCNL SIRS in patients with kidney stone

Variables	SIRS (Univariate analysis)			SIRS (Multivariate analysis)			MS (Univariate analysis)			MS (Multivariate analysis)		
	HR	95% CI	P value	HR	95% CI	P value	HR	95% CI	P value	HR	95% CI	P value
NLR	5.764	4.038∼8.227	<0.001	6.743	3.947∼11.518	<0.001	1.246	1.107∼1.402	<0.001	0.943	0.762∼1.168	0.593
dNLR	10.896	6.676∼17.782	<0.001	7.844	4.257∼14.455	<0.001	1.744	1.370∼2.220	<0.001	1.893	1.277∼0.861	0.225
LMR	0.571	0.492∼0.662	<0.001	0.995	0.803∼1.235	0.967	0.743	0.629∼0.877	<0.001	0.602	0.461∼0.785	<0.001
PLR	1.014	1.010∼1.018	<0.001	1.001	0.995∼1.007	0.781	1.007	1.003∼1.011	<0.001	1.005	0.998∼1.011	0.142
Age, (years)	1.003	0.986∼1.020	0.738	/	/	/	1.009	0.987∼1.031	0.426	/	/	/
Gender, (male%)	1.109	0.769∼1.601	0.579	/	/	/	0.930	0.579∼1.492	0.763	/	/	/
Residence, (urban)	0.760	0.527∼1.094	0.140	/	/	/	0.815	0.507∼1.311	0.400	/	/	/
BMI, (kg/m2)	0.981	0.925∼1.041	0.528	/	/	/	1.332	1.218∼1.455	<0.001	1.435	1.260∼1.633	<0.001
Hypertension	2.218	1.4518∼3.389	<0.001	1.665	0.898∼3.086	0.106	4.195	2.550∼3.902	<0.001	1.919	0.973∼3.786	0.060
Killip Level	1.465	0.761∼2.824	0.253	/	/	/	2.795	1.403∼5.566	0.003	1.278	0.483∼3.382	0.622
Diabetes	0.979	0.602∼1.592	0.932	/	/	/	8.987	5.282∼15.290	<0.001	8.858	3.636∼21.579	<0.001
ASA	0.916	0.679∼1.236	0.565	/	/	/	2.433	1.646∼3.596	<0.001	2.552	1.452∼4.485	<0.001
White blood cell, (10^9^/L)	1.326	1.182∼1.488	<0.001	1.339	1.168∼1.535	<0.001	1.201	1.047∼1.379	0.009	1.718	0.458∼6.447	0.423
Neutrophil, (10^9^/L)	2.072	1.747∼2.458	<0.001	3.131	2.305∼4.251	<0.001	1.416	1.214∼1.653	<0.001	0.806	0.166∼3.907	0.789
Lymphocyte, (10^9^/L)	0.190	0.125∼0.288	<0.001	0.081	0.041∼0.163	<0.001	0.450	0.285∼0.711	0.001	0.207	0.048∼0.891	0.034
Monocyte, (10^9^/L)	3.775	1.227∼11.620	<0.021	0.831	0.107∼6.423	0.859	1.462	0.446∼4.793	0.530	/	/	/
Platelet, (10^9^/L)	1.000	0.998∼1.003	0.814	/	/	/	1.003	0.999∼1.006	0.141	/	/	/
Hemoglobin, (g/L)	0.983	0.975∼0.991	<0.001	0.988	0.973∼1.004	0.144	1.007	0.996∼1.018	0.211	/	/	/
Alkaline phosphatase, (IU/L)	0.997	0.990∼1.005	0.511	/	/	/	0.091	0.979∼1.002	0.091	/	/	/
Albumin, (g/L)	0.949	0.912∼0.988	0.010	1.090	1.006∼1.181	0.035	0.977	0.929∼1.027	0.358	/	/	/
Total Cholesterol, (mmol/L)	0.778	0.6191∼0.979	0.033	0.836	0.600∼1.166	0.291	1.477	1.124∼1.941	0.005	1.586	1.093∼2.302	0.015
FBG, (mmol/L)	1.039	0.901∼1.199	0.595	/	/	/	1.690	1.387∼2.059	<0.001	1.179	0.902∼1.541	0.227
Sodium, (mmol/L)	1.002	0.959∼1.047	0.926	/	/	/	1.071	0.986∼1.165	0.106	/	/	/
Chloride, (mmol/L)	1.009	0.959∼1.061	0.731	/	/	/	0.998	0.935∼1.065	0.947	/	/	/
Calcium, (mmol/L)	0.205	0.053∼0.784	0.021	0.838	0.406∼1.732	0.634	1.089	0.982∼1.205	0.109	/	/	/
Potassium (mmol/L)	0.987	0.951∼1.024	0.480	/	/	/	0.988	0.937∼1.042	0.665	/	/	/
Bicarbonate, (mmol/L)	0.940	0.898∼0.984	0.008	0.915	0.846∼0.990	0.027	0.995	0.945∼1.047	0.838	/	/	/
Uric acid, (umol/L)	1.000	0.998∼1.001	0.854	/	/	/	1.001	0.999∼1.003	0.380	/	/	/
Serum creatinine, (umol/L)	1.001	1.000∼1.003	0.200	/	/	/	1.001	0.999∼1.002	0.263	/	/	/
BUN, (mol/L)	1.027	0.997∼1.059	0.082	/	/	/	1.022	0.986∼1.060	0.238	/	/	/
GFR, (ml/min)	0.991	0.985∼0.997	0.002	1.002	0.990∼1.013	0.780	0.995	0.987∼1.002	1.002	/	/	/
Stone burden, (mm^2^)	1.002	1.001∼1.003	<0.001	1.001	1.000∼1.002	0.133	1.000	0.999∼1.001	0.881	/	/	/
Operative time, (minutes)	1.004	1.001∼1.008	0.021	0.999	0.993∼1.005	0.783	1.008	1.003∼1.013	0.001	1.010	1.004∼1.016	0.002
Stay length, (days)	1.098	1.063∼1.134	<0.001	1.072	1.021∼1.125	0.005	1.030	0.996∼1.065	0.083	/	/	/
Multiple nephrolith	4.020	2.008∼8.048	<0.001	2.988	1.039∼8.59	0.042	0.642	0.348∼1.184	0.156	/	/	/
Staghorn nephrolith	2.127	1.351∼3.347	0.001	1.052	0.505∼2.194	0.892	1.181	0.656∼2.125	0.579	/	/	/
Hydronephrosis	8.512	3.6260∼19.983	<0.001	16.288	4.332∼61.234	<0.001	0.620	0.345∼1.114	0.110	/	/	/

NLR, neutrophil to lymphocyte ratio; dNLR, neutrophil to (white cell count- neutrophil count) ratio; LMR, lymphocyte to monocyte ratio; PLR, platelet count to lymphocyte ratio; BMI, body mass index; ASA, American Society of Anesthesiologists; FBG, fasting blood-glucose; BUN, blood urea nitrogen; GFR, glomerular filtration rate, SIRS, systemic inflammatory response syndrome; MetS, metabolic syndrome.

Additionally, via univariate Cox proportional hazards analysis factors associated with post-PCNL SIRS included the NLR, dNLR, LMR, PLR, gender, hypertension, Killip level, white blood cell, neutrophil, lymphocyte, monocyte counts, hemoglobin, total cholesterol, bicarbonate, GFR, stone burden, operative time, stay length, multiple nephrolith, staghorn nephrolith and hydronephrosis (all P < 0.05). After extensive univariate analysis, these significant variables were included in the multivariable Cox proportional hazards models, which showed that the level of NLR (HR 6.743, 95%CI 3.947∼11.518), dNLR (HR= 7.844, 95%CI 4.257∼14.455), White blood cell (HR 1.339, 95%CI 1.168∼1.535), Neutrophil (HR 3.131, 95%CI 2.305∼4.251), Lymphocyt (HR 0.081, 95%CI 0.041∼0.163), Albumin (HR 1.090, 95%CI 1.006∼1.181), Bicarbonat (HR 0.915, 95%CI 0.846∼0.990), Stay length (HR 1.072, 95%CI 1.021∼1.125), Multiple nephrolith (HR 2.988, 95%CI 1.039∼8.59) and Hydronephrosis (HR 16.288, 95%CI 4.332∼61.234) were significant predictors of post-PCNL SIRS.

## DISCUSSION

Nephrolithiasis is a common disease whose recurrence rates are up to 50% and 80% within the first five and ten years, respectively, after an initial stone episode. To our knowledge, this is the first research to evaluate NLR, dNLR, LMR and PLR as the predictors of kidney stone. Our study indicates, a significant correlation between inflammation biomarkers and kidney stones. Patients with kidney stone have higher NLR, dNLR, LMR and PLR levels than healthy controls. Additionally, patients with MetS, post-PCNL SIRS have higher NLR, dNLR, LMR and PLR levels than those without. We also demonstrated that increased pre-operation NLR, dNLR, LMR and PLR positively correlated with MetS comorbidities and post-PCNL SIRS in patients with kidney stones. The Cox regression multivariate analyses indicated that NLR appeared to be potentially independent useful inflammatory biomarker of in patients with kidney stone, and could serve as a new inflammatory markers for monitoring disease activity, especially for patients with kidney stones in critical care following PCNL. Although dNLR, PLR and LMR were significantly associated with survival in univariate analysis, they were not maintained as independent indicators in the multivariate model. These results were supported by several mechanisms of inflammatory reactions to kidney stones.

MetS has been recognized as one of the most relevant clinical components associated with kidney stone. It is known that MetS is associated with systemic inflammation and in patients with MetS, the risk for development of kidney stone increases. In patients with kidney stones and metabolic syndrome who had PCNL. Also, MetS was associated with deterioration of renal function in the long-term follow-up, [26] The estimated prevalence of MetS in patients with kidney stone is 4.7% to 20.7%, [27, 28] and the prevalence of MetS in our study is 16.2%. In the present study, we reported that inflammatory markers NLR, dNLR, LMR and PLR were higher in kidney stone patients with than without MetS comorbidities. This result indicates that the presence of MetS in patients with kidney stones is associated with more intense systemic inflammation. The results may have clinical importance, because the inflammation factors indicating MetS in kidney stone may be early markers of developing cardiovascular events.

Previous researches have reported that even with careful preparation before operation, SIRS occurs in 11%-35% of patients following PCNL and progress to sepsis in 2.5% of patients [29, 30]. In our study, the total SIRS occurrence rate was 35.3% in patients with larger stones and obstructed pelvicalyceal system, consistent with findings reported by Bag et al with the risk of 49% [31].

The relationship between elevated pre-operative NLR, dNLR, LMR and PLR and kidney stones is yet to be elucidated. Firstly, neutrophil was triggered by stone-stimulated inflammatory factors, including the granulocyte colony stimulating factor, tumor necrosis factor-alpha, interleukin-6, and myeloid growth factors. Moreover, elevated circulating neutrophils have been reported prompt secretion of inflammatory mediators and therefore accelerate stone formation. Thus elevated neutrophils may aid in the formation of kidney stone by providing an adequate environment. Secondly, decreased lymphocyte count may also play an important roles in inflammatory reaction to stones. Known as an inflammatory response, neutrophil suppresses the immune response by inhibiting the cytolytic activity of immune cells such as lymphocytes, activated T cells, and natural killer cells [32].

There is an intimate relationship between kidney stone and inflammatory cytokines. Extrapolating this to the microenvironment of the kidney, some investigators of pro-inflammatory state have found in the presence of molecules generally involved in inflammatory pathways, such as osteopontin, heavy chain of inter-alpha-inhibitor, collagen, and the zinc in the nephron, specifically in interstitial plaques of the renal papillae in stone formers [33, 34]. Reactive oxygen species (ROS) and oxidative stress (OS) represent well-known factors that contribute to the progress of MetS and cardiovascular diseases [35, 36].

Finally, neutrophils play an important role both in pro- and anti-tumor functions in cancer and immune-related disease progression. In two recent publications in Nature, Coffelt *et al.* reveal a novel mechanism that neutrophils and T cells cooperate to generate a metastasis-supporting immune suppression, and Wculek *et al.* found that neutrophils have a fundamental role in inflammatory responses and their contribution to tumorigenesis [37]. These findings support the conclusion that elevated NLR could predict poor survival in cancer patients, and thus suggested to us that neutrophils may also play a similar role in the kidney stone formation and progression.

The possible advantage of this study was that it focused on the clinical utility of this type of laboratory biomarkers. For the kidney stone patients with higher risk of post-PCNL after evaluating preoperative NLR, careful preparation peri-operation and post-operative standard-of-care were recommended. It also raised the cost-effectiveness of routine blood test.

The current study has several limitations that should to be taken in account. Firstly, our results were from a retrospective design, single-center study and thus the predicative significance of systematic inflammatory biomarkers in patient’s with kidney stones remains to be confirmed by prospective and clinical validation studies. Although we demonstrated the initial evidence that NLR, dNLR, LMR and PLR were increased in patients with kidney stone which were associated with post-PCNL SIRS and MS comorbidities, the NLR, dNLR, LMR and PLR were not predictors of kidney stone formation. Besides further basic studies will be carried out to identify the detailed mechanisms by which kidney stones mediate inflammatory cells responses and inflammatory mediators stimulated kidney stone formation and accumulation. Finally, since CRP and PCT are not routinely tested in our clinical practice, therefore CRP and PCT were not included in our analyses. Further researches should compare the predictive role between NLR, dNLR and CRP, PCT as well as clinicopathological parameters.

In conclusion, we demonstrate for the first time that NLR, dNLR, LMR and PLR are all increased in kidney stone patients, and positively correlated with MetS comorbidities and post-PCNL SIRS. In multivariate analysis, NLR appears to be potentially useful inflammatory biomarkers of systemic inflammation in patients with kidney stone, which suggests that increased NLR level before surgery was independent and adverse predictor of MS comorbidities and post-PCNL SIRS. We suggest that clinicians should consider the level of NLR before surgery to help select the most appropriate therapy plan for their patients with kidney stone, especially for operational evaluation in MetS comorbidities patients and monitoring the SIRS following PCNL.

## MATERIALS AND METHODS

### Study population

Retrospective investigation and analysis were carried out in patients with kidney stones who had PCNL at Tongji Hospital between January 2015 and December 2015. The inclusion criteria were as follows: 1) A CT and stone composition analysis was performed for all patients with a kidney stone; 2) Treated with PCNL; 3) No history of previous therapies or other malignancies; 4) No perioperative mortality; 5) No antibiotic treatment before surgery, infection and hyperpyrexia; 6) Preoperative blood parameter data available; 7) Informed consents were obtained from eligible patients. Exclusion criteria included: a previous placement of stent, nephrostomy tube or indwelling catheter, history of renal failure, fever prior to surgery, presence of infectious diseases on admission, previous interventional treatment, concomitant bladder stone or tumor, concomitant therapy with other antibiotics, the presence of contra-lateral renal or ureteral stone, and complicated with cyst and malignancy. Through this approach we successfully recruited 513 patients with kidney stones and 204 healthy controls in our study.

### Data collection

For each patient and healthy control, we collected the following clinical and pathological information: age, gender, BMI, residence, hypertension, Killip level, diabetes, ASA score, stone characteristics (stone burden, multiple nephrolith, staghorn nephrolith and hydronephrosis) and perioperative variables (operation time and stay length and post-PCNL SIRS rates). All patients were performed routine blood tests (white blood cell, neutrophil, lymphocyte, monocyte, platelet counts, hemoglobin, alkaline phosphatase, albumin, total cholesterol, FBG, sodium, chloride, calcium, potassium, bicarbonate, BUN, uric acid, serum creatinine and GFR) and imaging test (plain radiography of kidneys, ureters and bladder (KUB), renal ultrasonography, ntravenous urography (IVU) or computed tomography (CT). Clinical parameters such as hydronephrosis were found by physical examination and confirmed by ultrasonography, CT or magnetic resonance imaging (MRI). Stone characteristics included the number of stones (detected via CT or MRI scan), staghorn (yes/no), stone location and stone burden. Stone surface area (stone burden) was calculated by the following formula: length × width × π × 0.25 [38]. In cases of multiple calculi, stone burden was defined as the sum of the surface area of each stone.

### Definition

Hematological parameters were obtained 1 week prior to kidney stone treatment. Preoperative NLR, dNLR, LMR and PLR were measured by peripheral blood cell count, and were described as follows: NLR = neutrophil to lymphocyte ratio; dNLR = neutrophil to (white cell count- neutrophil count) ratio; LMR = lymphocyte to monocyte ratio; and PLR = platelet count to lymphocyte ratio. MetS was defined as: individuals with 3 out of the 5 criteria of the National Cholesterol Education Program Adult Treatment Panel III (NCEP ATP III) were met, modified for pre-diabetes (fasting glucose 100-125 mg/dL) [39]. In the post-operative period, patients were monitored for signs of SIRS, as defied by the development of 2 of 5 criteria:(1) temperature less than 36 or over 38, (2) heart rate more than 90 beats per minute, (3) tachypnea more than 20 breaths per minute, (4) l12×10 [9]/L or more than 4×10^9^/L, (5) A systolic blood pressure below 90 mmHg or decrease of 40 mmHg below baseline in the presence of SIRS was defied as septic shock [40].

### Ethics

This study was approved by the Medical Ethics Committee of Huazhong University of Science and Technology. Written informed consent was obtained from all participants.

### Statistical analyses

Statistical analyses was performed using GraphPad Prism version 5.013 (San Diego, CA, USA), SPSS 2.0 (IBM Corporation, Armonk, NY, USA) and R software version 3.2.0 (http://www.r-project.org/). The optimal cut-off levels of NLR, dNLR, LMR, and PLR were determined by receiver operating curve (ROC) analysis. Associations of NLR, dNLR, PLR, and LMR with other clinicopathological factors were determined using student’s t-test, chi-square test and linear-regression analysis. Univariate and multivariate analyses were determined by the Cox regression model. Variables that reached a level of statistical significance in the univariate analysis were included into the multivariable analysis. All statistical tests were two sided and p-values less than 0.05 (p < 0.05) were considered statistically significant unless otherwise specified.
